# The effect of synbiotics *Bifidobacterium infantis* and milk oligosaccharides on shaping gut microbiota community structure and NASH treatment

**DOI:** 10.1016/j.dib.2018.05.127

**Published:** 2018-05-24

**Authors:** Prasant Kumar Jena, Lili Sheng, Nidhi Nagar, Chao Wu, Daniela Barile, David A. Mills, Yui-Jui Yvonne Wan

**Affiliations:** a Department of Pathology and Laboratory Medicine, University of California, Davis, Sacramento, CA, USA; b Research and Development, Hilmar Ingredients, Hilmar, CA, USA; c Department of Food Science and Technology, University of California, Davis, CA, USA; d Department of Viticulture and Enology, University of California, Davis, CA, USA

**Keywords:** Probiotics, Western diet, Short chain fatty acid, Inflammation

## Abstract

Probiotic *Bifidobacterium longum* subspecies *infantis* (*Bifidobacterium infantis*) consumes human milk oligosaccharides (MO) and protects intestinal permeability thereby having anti-inflammatory effects (Underwood et al., 2015; Bode, 2006; Asakuma et al., 2011) [1–3]. Via the gut-liver axis, gut barrier disruption and dysbiosis lead to hepatic inflammation (Sheng et al., 2017; Jena et al., 2017) [4,5,6]. Our published data revealed that butyrate, as well as synbiotics of *B. infantis* in combination with MO, had protective effects against cancer-prone non-alcoholic steatohepatitis (NASH) mouse models, i.e., Western diet (WD)-fed bile acid receptor FXR (farnesoid x receptor) knockout (KO) mice (Jena et al., 2018) [6,7]. In addition, MO was particularly effective in increasing the blooming of butyrate-generating bacteria (Jena et al., 2018) [7]. In the present study, we further showed that the reduced ileal short chain fatty acid (SCFA) signaling found in WD-fed FXR KO mice could be reversed by *B. infantis* and/or MO treatment. Moreover, ileal mRNA levels of SCFA receptors i.e. *Gpr41* (*Ffar3*), *Gpr109* (*Hcar2*), and *Gpr43* (*Ffar2*) were increased in *B. infantis* and/or MO-treated mice suggesting increased SCFA signaling (Fig. 1). Further, nuclear magnetic resonance (NMR) data revealed that MO and *B. Infant*is plus MO increased intestinal acetate, propionate, butyrate, and valerate levels (Fig. 2). In addition, *B. infantis* and/or MO reduced the abundance of genus *Bilophila* and the relative copy number of bacterial genes including dissimilatory sulfite reductase (*dsrA*) and methyl coenzyme M reductase A (*mcrA*), which were all increased in cancer-prone FXR KO mice (Fig. 3).

**Specifications Table**

**Table:** 

Subject area	*Biology, Microbiology, Molecular Biology*
More specific subject area	*Synbiotics*
Type of data	*Table, figure, graph*
How data was acquired	*Real time PCR, NMR*
Data format	*Analyzed*
Experimental factors	*C57BL/6 wild type and FXR KO male mice were fed with Western diet after weaning. When mice were 3-month old, they were given B. infantis (10*^*9*^*cfu per mouse, orally, once a week) in saline, bovine MO (7% in a diet that lacks 7% cellulose), or a combination of B. infantis plus MO while mice continued a WD for 7 months and euthanized when they were 10-month old.*
Experimental features	*Gene expression in ileum and SCFA concentration in cecal content were measured by real time PCR and NMR, respectively.*
Data source location	*Sacramento, California, United States of America*
Data accessibility	*Data with this article*

**Value of the data**

– In our earlier report, *B. infantis* and MO treatment reduced hepatic and ileal inflammation [7]. It has been shown that *B. infantis* and MO protects from gut barrier function and inflammation [1], [2], [3]. In addition, the abundance of bacterial butyrate-generating genes *bcoA* (butyryl-CoA: acetate CoA-transferase) and *buk* (butyrate kinase) in the cecum was increased with MO and *B. infantis* plus MO treatment. Data in this study described the mRNA expression of SCFA receptors regulated by *B. infantis* and MO treatment. Our data showed that increased SCFA signaling with *B. infantis* and MO treatment was associated with reduced inflammation.
– In our earlier report, the concentration of butyrate was reduced in FXR KO mice [6]. Data in this study described that *B. infantis* and MO increased SCFAs, i.e., acetate, propionate, butyrate, and valerate. Additionally, such increases were accompanied by reduced inflammation in FXR KO mice. It has been shown that dysregulated bile acid and gut dysbiosis causes hepatic inflammation [4], [5], [6], [7]. Moreover, the abundance of genus *Bilophila* and *dsrA* gene, which are involved in the generation of hydrogen sulfide, was increased by FXR inactivation and *B. infantis* and MO treatment reversed those changes.
– These data suggested that the anti-inflammatory effect of *B. infantis* and MO may due to increased SCFAs and reduced hydrogen sulfide and methane.

## 1 Data

**Fig. 1:**
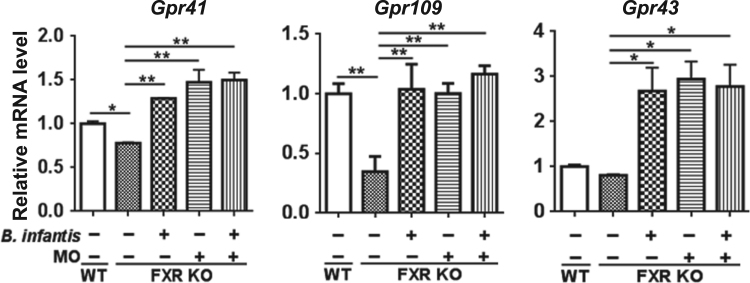
The effects of *B. infantis* and/or MO on SCFA receptor signaling. Ileal mRNA level of indicated genes in WD-fed WT mice and WD-fed FXR KO mice supplemented with and without *B. infantis* and/or MO for 7 months. Data expressed as mean ± SD. *n* ≥ 6 per group. *p< 0.05, **p< 0.01. WT mice compared with FXR KO mice, and untreated FXR KO mice compared with treated FXR KO mice on the same diet.

**Fig. 2:**
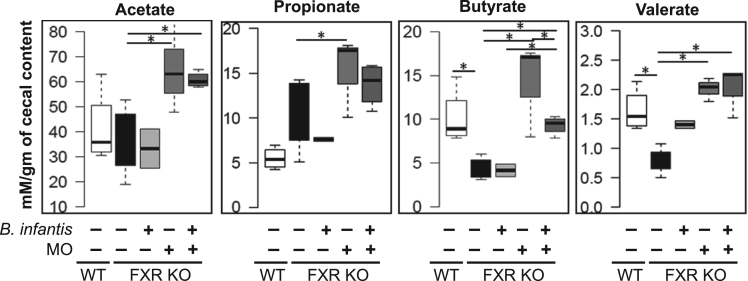
NMR analysis of SCFA concentration in cecal content of WD-fed WT mice and WD-fed FXR KO mice treated with and without *B. infantis* and/or MO. Data expressed as mean±SD. *n* ≥ 6 per group. *p< 0.05. WT mice compared with FXR KO mice, and untreated FXR KO mice compared with treated FXR KO mice.

**Fig. 3:**
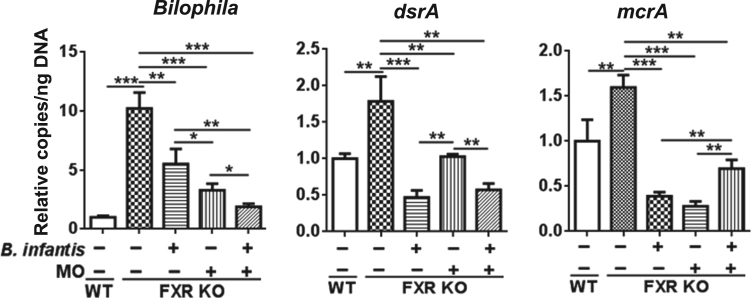
Targeted functional quantitative PCR analyses of microbial genes in WD-fed WT mice and WD-fed FXR KO mice supplemented with and without *B. infantis* and/or MO. Data expressed as mean±SD. *n* ≥ 6 per group. *p< 0.05, **p< 0.01, ***p< 0.001, WT mice compared with FXR KO mice, and untreated FXR KO mice compared with treated FXR KO mice.

The data showed the mRNA level of ileal SCFA receptors i.e. *Gpr41*, *Gpr109a*, and *Gpr43* in WD-fed wild type (WT) mice and WD-fed FXR KO mice supplemented with or without *Bifidobacterium infantis* and/or MO (Fig. 1). NMR data generated from cecal content showed the concentration of acetate, propionate, butyrate, and valerate (Fig. 2). Moreover, bacteria and their functional genes were determined by real-time quantitative PCR (Fig. 3).

## 2 Experimental design

### 2.1 Mice

Specific pathogen-free male C57BL/6 WT mice (Jackson Laboratory, Sacramento, CA, USA) and FXR KO mice were housed in steel microisolator cages at 22 °C with a 12-h light/dark cycle. They were given a WD containing 21.2% fat, 34% sucrose, and 0.2% cholesterol (Envigo, Indianapolis, IN, USA) right after weaning (3 weeks, 6–10 mice per group). When mice were 3-month old, they were given *B. infantis* (10^9^ cfu per mouse, orally, once a week) in saline, bovine MO (7% in a diet that lacks 7% cellulose), or a combination of *B. infantis* plus MO while mice continued a WD until they were 10-month old. Experiments were conducted in accordance with the National Institutes of Health Guidelines for the Care and Use of Laboratory Animals under protocols approved by the Institutional Animal Care and Use Committee of the University of California, Davis.

### 2.2 Gene expression and quantification of bacterial genes

RNA was isolated from mouse tissues using TRIzol (Invitrogen, Carlsbad, CA, USA) and reverse transcribed into cDNA. qRT-PCR was performed on an ABI 7900HT Fast real-time PCR system using Power SYBR Green PCR Master Mix (Applied Biosystems, Foster City, CA, USA). The mRNA levels were normalized to the level of *Gapdh* mRNA. The primer sets used are described in Table 1.

**Table 1:** Primers used for qPCR.

Gene		Forward (5’ – 3’)	Reverse (5’ – 3’)
qPCR primers used to quantify gene expression in mice			
*Gpr41*		GTGACCATGGGGACAAGCTTC	CCCTGGCTGTAGGTTGCATT
*Gpr109a*		ATGGCGAGGCATATCTGTGTAGCA	TCCTGCCTGAGCAGAACAAGATGA
*Gpr43*		GGCTTCTACAGCAGCATCTA	AAGCACACCAGGAAATTAAG
qPCR primers used to quantify bacterial genes			
*Bilophila (tpA)*		CGGTATCGAAATCGTGAAGG	CAGAGGGTCAGGGTGTTGTT
*dsrA*		GCCGTTACTGTGACCAGCC	GGTGGAGCCGTGCATGTT
*mcrA*		TTCGGTGGATCDCARAGRGC	GBARGTCGWAWCCGTAGAATCC

For bacterial gene quantification, cecal (0.05 g) DNA was extracted using ZR Fecal DNA MiniPrep Kit (Zymo Research, Irvine, CA, USA), quantified by NanoDrop (Thermo Scientific, Wilmington, DE, USA), and amplified using primers listed in Table 1. A dissociation step was included to analyze the melting profile of amplified products. In parallel, qPCR was performed using ten-fold serial diluted synthetic DNA fragments (Integrative DNA technologies, Redwood city, CA, USA) of a bacterial gene with known concentrations. Bacterial DNA concentration was calculated using standard curves of diluted synthetic DNA fragment based on method described elsewhere [8].

### 2.3 ^1^H NMR spectroscopy for metabolic profiling

Cecal samples (50−60 mg) were prepared and analyzed by ^1^H NMR based on published method [6], [9]. Each cecal sample was homogenized and mixed with sodium-potassium phosphate buffer and centrifuged at 14,000*g* for 20 min. All ^1^H NMR spectra were collected using a Bruker advance 600 NMR spectrometer (Bruker; Rheinstetten, Germany). The samples were pre-cooled to 277 K before being loaded into the magnet, and were warmed to 298 K and equilibrated for 5 min before data acquisition. For each sample, 64 scans were collected into 64 K data points over a spectral width of 20 ppm with a relaxation delay of 2 s. Identification and quantification of metabolites were accomplished using Chenomx NMR Suite 7.6 (Chenomx, Inc., Edmonton, Alberta, Canada).

### 2.4 Statistical analysis

Data are presented as the mean ± SD. Statistical analysis was performed by unpaired Student׳s *t*-test or one-way analysis of variance by using GraphPad Prism 6.0 (GraphPad, La Jolla, CA, USA). *P* < 0.05 was considered statistically significant.
