# Supplementary material for: The effect of synbiotics Bifidobacterium infantis and milk oligosaccharides on shaping gut microbiota community structure and NASH treatment
Source: Data Brief. 2018 May 24;19:1025–9. doi: 10.1016/j.dib.2018.05.127 (PMC5997954; doi:10.1016/j.dib.2018.05.127)
Supplement: Supplementary file 1 — Supporting information [file mmc1.docx]

**Competing interests**

The authors of this paper declare that they do not have any disclosures regarding funding or conflict of interest.
